# Evaluation of the Panbio™ COVID-19 ag rapid test at an emergency room in a hospital in São Paulo, Brazil

**DOI:** 10.1016/j.bjid.2022.102349

**Published:** 2022-03-21

**Authors:** Klinger Soares Faíco-Filho, Francisco Estivallet Finamor Júnior, Luiz Vinícius Leão Moreira, Paulo Ricardo Gessolo Lins, Alberto Fernando Oliveira Justo, Nancy Bellei

**Affiliations:** a Universidade Federal de São Paulo (UNIFESP), Escola Paulista de Medicina (EPM), Laboratório de Virologia, Departamento de Medicina, Divisão de Doenças Infecciosas, São Paulo, SP, Brazil; b Universidade Federal de São Paulo (UNIFESP), Departamento de Medicina, Disciplina de Medicina de Emergência, São Paulo, SP, Brazil; c Universidade Federal de São Paulo (UNIFESP), Departamento de Medicina, Disciplina de Nefrologia, São Paulo, SP, Brazil

**Keywords:** SARS-CoV-2, Panbio™ COVID-19 Ag test, Hospital

## Abstract

**Background:**

The performance characteristics of the Panbio™ COVID-19 Ag test was evaluated at an emergency room setting against RT-PCR, considered the gold-standard for the detection of SARS-CoV-2, in São Paulo, Brazil. The study aimed to determine the sensitivity, specificity, Positive Percent Agreement (PPA), and Negative Percent Agreement (NPA) as compared to RT-PCR.

**Methods:**

Specimens from 127 suspected patients were tested by both the Panbio™ COVID-19 Ag test and by RT-PCR.

**Results:**

In relation to RT-PCR using Ct values ≤ 40 as the upper limit for positivity, the Panbio™ COVID-19 Ag test showed an overall sensitivity of 84.3% (95% CI 75‒93.8%) and 98.2% (95% CI 96‒98.8%) overall specificity. For Ct values ≤ 25 (*n* = 37), the Panbio™ COVID-19 Ag test showed 97% sensitivity.

**Discussion:**

The concordance between the Panbio™ COVID-19 Ag test and RT-PCR was 97% at Ct values below 25 but decreased at higher Ct values. For disease control, it is very important to identify infected individuals who present COVID-19 symptoms and also those who are suspected of infection due to contact with infected individuals.

**Conclusion:**

The Panbio™ COVID-19 Ag test is suitable for use as a diagnostic test for rapid screening of patients presenting COVID-19 symptoms, or those suspected of being infected, prior to being admitted to hospital.

## Background

In late 2019, an outbreak of respiratory illness of unknown etiology was reported in Wuhan City, Hubei Province, China. The International Committee for Taxonomy of Viruses (ICTV) named the virus SARS-CoV-2 and is the cause of the global COVID-19 pandemic which, as of 18th February 2022, has claimed more than 5.8 million lives (https://covid19.who.int/). The preferred diagnostic test for COVID-19 is Nucleic Acid Amplification Test (NAAT) using Reverse Transcriptase Polymerase Chain Reaction (RT-PCR) performed in fluid from the nasal or nasopharyngeal cavities. The turn-around time can vary from same one day to one week.^1^, ^2^, ^3^ Alternatively, a COVID-19 antigen test using nasal or nasopharyngeal samples can produce results in minutes.^4^ A positive antigen test result is considered accurate, but low viral load in the sample can produce false-negative results.^5^^,^^6^

The Abbott Panbio™ COVID-19 Ag Rapid Test device is an *in vitro* rapid diagnostic test intended to aid in the diagnosis of COVID-19. The Panbio™ COVID-19 Ag Rapid Test device is a lateral flow immunochromatographic test used for the qualitative detection of SARS-CoV-2 antigens in human tissue fluids obtained from nasal or nasopharyngeal swabs. The Panbio™ COVID-19 Ag platform uses a cassette containing a lateral flow test strip and is intended for use by trained healthcare professionals in point-of-care and hospital settings. The product may be used in both laboratory and non-laboratory environments that meet the requirements specified in the product's Instructions For Use (IFU).

The Panbio™ COVID-19 Ag test can be used for rapid screening in patients presenting COVID-19 symptoms, or in those who had contact with infected persons, prior to being admitted to hospital. In this study, the diagnostic performance, namely sensitivity, specificity, positive and negative predictive values, of the Panbio™ COVID-19 Ag Rapid test device using fluids obtained from nasopharyngeal swabs was assessed having RT-PCR test as gold-standard.

## Methods

### Study population

This cross-sectional study was conducted at São Paulo Hospital, São Paulo, Brazil. Patients (≥18 years), treated at the emergency room and admitted for at least 24 hours were included if they met one of the following criteria: (1) symptoms suspected to be related to SARS-CoV-2 and/or contact with COVID-19 infected persons, (2) decompensation of underlying disease, or (3) suggestive tomographic alteration (ground glass opacities).

### Testing scheme

Nasopharyngeal swab samples were simultaneously tested using the Panbio™ COVID-19 Ag test and by RT-PCR. The Panbio™ COVID-19 Ag test was performed according to the product Instructions For Use (IFU).^7^ Results were obtained and interpreted at 15 minutes after test initiation according to the IFU. The RT-PCR testing was performed with GeneFinder™ COVID-19 Plus RealAmp Kit (OSANG Healthcare Co., Ltd.) according to manufacturer's instructions targeting the *RdRp* (RNA-dependent RNA Polymerase), *E* (Envelope), and *N* (Nucleocapsid) SARS-CoV-2 genes.^8^ Samples with inconclusive GeneFinder™ test results were tested using a second RT-PCR test – Mobius XGEN MASTER COVID-19 test – targeting the *ORF1ab* and *N* SARS-CoV-2 genes, per the IFU.^9^ Test results for both RT-PCR methods were available within 6‒24 hours.

### Reference testing

For this study, the performance of the Panbio™ COVID-19 Ag test was evaluated against the results of the gold standard test (RT-PCR). For RT-PCR, RNA was isolated from the subject's nasopharyngeal swab using the Quick-RNA Viral Kit (Zymo Research, Irvine, CA), according to the manufacturer's protocol.^10^ After extraction, the RNA was used immediately, and the remaining RNA was stored at -80°C. The Master Mixture was prepared by mixing 10 µL of COVID-19 Plus Reaction Mixture and 5 µL of COVID-19 Plus Probe Mixture per sample; a sufficient amount of Master Mixture was prepared for all the samples and controls that were tested. Then, 15 µL of the Master Mixture was transferred to a 96-well plate to which either (1) 5 µL RNA sample, (2) 5 µL negative control (DEPC-treated water), or (3) 5 µL positive control (DNA plasmids encoding the SARS-CoV-2 *RdRp, E*, and *N* genes, and human *RNase P* gene). The plate was sealed and centrifuged at 2,000 rpm for 10 seconds. The thermal cycling conditions were as follows: (1) 1 cycle at 50°C for 20 min, (2) 1 cycle at 95°C for 5 min, and (3) 45 cycles at 95°C for 15 sec and 58°C for 60 sec.

In contrast to the GeneFinder™ kit, the Master Mixture in the Mobius™ kit was used directly without further preparation. The 96-well plate was prepared by adding 15 µL of the Master Mixture to the sample wells, followed by either (1) 5 µL of the subject's RNA, (2) 5 µL negative control (DEPC-treated water), or (3) 5 µL positive control (a synthetic cDNA of the *ORF1ab* and *N* SRS-CoV-2 genes). In addition, 1 µL of an internal control was added to all the subjects’ RNA samples. It is important to highlight that the content of the internal control is not described by the manufacturer. The plate was sealed and centrifuged, as described above. The thermal cycling conditions were as follows: (1) 1 cycle at 45°C for 15 min, (2) 1 cycle at 95°C for 2 min, and (3) 45 cycles at 95°C for 10 sec and 60°C for 50 sec.

### Statistical analysis

To assess differences between patient subgroups, continuous variables are expressed by mean (and standard deviation) or median (and interquartile range) values and compared using the Student *t*-test, ANOVA, Kruskal-Wallis test and/or the Mann-Whitney *U* test, as indicated. Categorical variables are presented as proportions and the respective 95% Confidence Intervals (95% CI) were compared using the Chi-Square test and the Fisher exact test.

The impact on sensitivity and specificity of the Panbio^TM^ COVID-19 Ag test according to the Ct value used to define a positive RT-PCR test was assessed by a Receiver Operating Characteristic (ROC) curve. The Area Under the Curve (AUC) and 95% Confidence Interval (95% CI) were calculated for Ct cutoffs of 25, 30, 35 and 40. All analyses were performed using the SPSS 26 program. Differences were considered statistically significant at p-values lower than 0.05.

The agreement beyond chance between results of RT-PCR, defining positivity according to the above Ct cutoffs, and the Panbio^TM^ COVID-19 Ag test was assessed by the kappa statistic.

## Results

A total of 127 subjects were included. The median age was 63 years (22‒69) and there were more males (54.3%) than females (vs. 45.7%). The characteristics of the study population are given in Table 1.

**Table 1.** RT-PCR results and clinical characteristics of study population^a^.

	PCR negative (*n* = 57)	PCR positive (*n* = 70)	p-value	Total (*n* = 127)
Age (mean ± SD)	54.74 ± 19.25	64.31 ± 14.83	0.002	60 ± 17.5
Male sex (%)	29 (50.9%)	40 (57.1%)	0.481	69 (54.3%)
Time of symptoms (days, mean)	5 (3; 7)	6 (4; 7)	0.06	5 (4; 7)
Ct value (mean, range)	40 (40; 40)	25 (21.75; 28)	< 0.001	32 (25; 40)

a Cutoff for PCR positivity Ct values ≤ 40.

The Panbio^TM^ COVID-19 Ag testing summary is shown in Table 2.

**Table 2.** Panbio^TM^ COVID-19 ag test results and clinical characteristics of study population.

	Antigen negative (*n* = 67)	Antigen positive (*n* = 60)	p-value	Total (*n* = 127)
Age (mean ± SD)	55.94 ± 18.60	64.57 ± 15.19	0.005	60 ± 17.5
Male sex (%)	35 (52.2%)	34 (56.7%)	0.617	69 (54.3%)
Time of symptoms (days, mean)	5 (3; 7)	6 (4; 7)	0.178	5 (4; 7)
Ct Value (mean, range)	40 (40; 40)	24.5 (21; 28)	< 0.001	32 (25; 40)

Table 3 provides details of the 10 samples that were negative by the Panbio^TM^ COVID-19 Ag test but were positive by RT-PCR using the GeneFinder™ COVID-19 Plus RealAmp Kit. The Ct values of the samples ranged from 21‒34 and were collected from subjects 2‒11 days post onset of symptoms.

**Table 3.** Details of discordant samples.

Sample	Ct value	Days post onset of symptoms
1	21	8
2	26	2
3		4
4		7
5	28	10
6	30	7
7	31	11
8	32	4
9 and 10	34	5
		6

Table 4 shows the diagnostic parameters sensitivity, specificity and positive and negative predictive values as a function of Ct. The results indicate that the Panbio^TM^ COVID-19 Ag test has high sensitivity (97.3%) when positivity was defined as a value ≤ 25, which falls to 84% when RT-PCR was considered positive for Ct values ≤ 40.

**Table 4.** Sensitivity, specificity, and positive and negative predictive values of Panbio^TM^ COVID-19 Ag test as a function of Ct

Panbio^TM^ COVID-19 Ag test	≤ 25	≤ 30	≤ 35	≤ 40
Positive	36	52	57	59
Negative	1	7	11	11
Sensitivity (%)	97.3	88.1	83.8	84.3
PPV (%)	60.0	86.7	95.0	98.3
NPV (%)	98.5	89.5	83.6	83.6
Accuracy (%)	80.3	81.2	89.0	90.6
Kappa	0.78	0.76	0.59	0.27

PPV, Positive Predictive Value; NPV, Negative Predictive Value.

The specificity of the Panbio^TM^ COVID-19 Ag test was 98.2% when all samples that were negative by RT-PCR were included (Ct values > 40). The accuracy of the test was 90.6%.

**Fig. 1:**
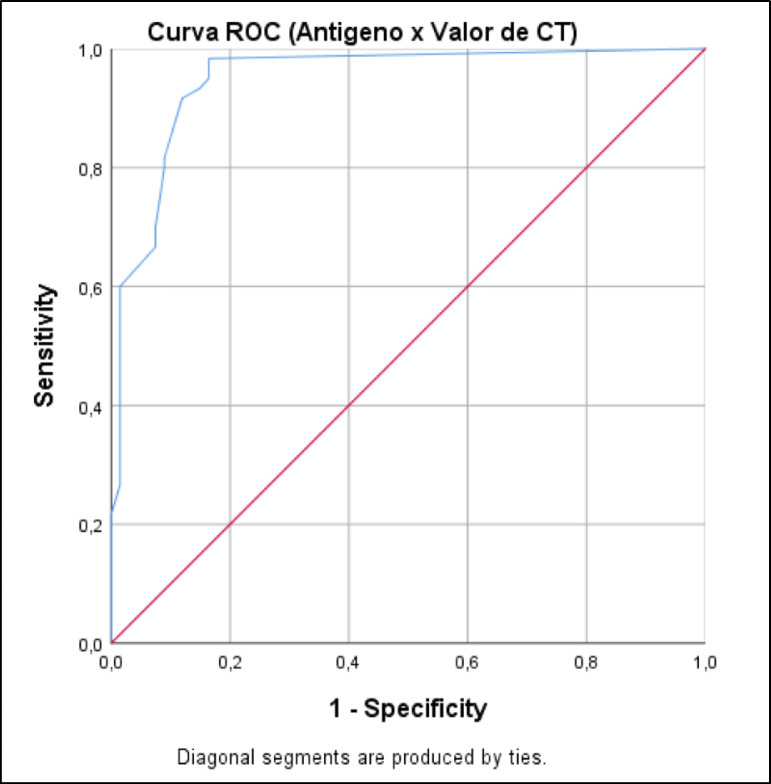
ROC curve of Panbio^TM^ COVID-19 Ag test results according to the Ct value used to define a positive RT-PCR test result.

Fig. 1 shows the ROC curve with the impact on sensitivity and specificity of the Panbio^TM^ COVID-19 Ag test generated by varying the defition of a real positive RT-PCR result taking into account the Ct value. The curve shows that the Ct cutoff of 25 gives the highest sensitivity (97.3%), NPV (98.5%) and AUC (0.949) of the Panbio^TM^ COVID-19 Ag test.

**Fig. 2:**
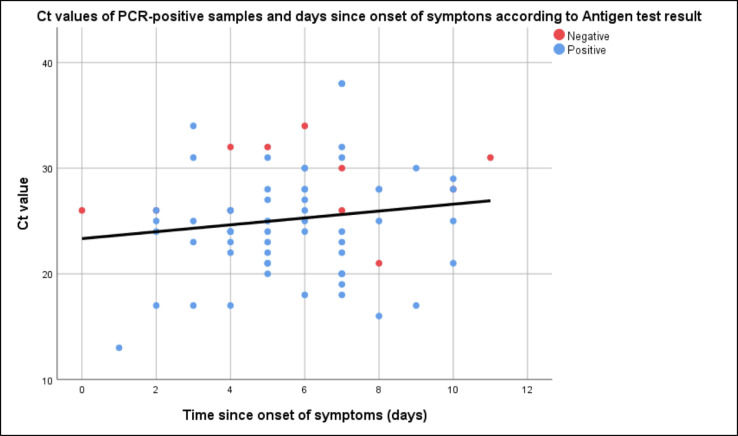
Distribution of Ct values according to time elapsed from symptoms onset among RT-PCR-positive samples.

The distribution of Ct values according to time elapsed from symptoms onset among RT-PCR-positive samples (*n* = 70) is shown in Fig. 2.

The number of data points is less than 70 because some samples produced the same Ct values and had the same number of days since symptom onset. No PCR-negative samples are presented here. The red squares represent PCR-positive samples that were tested as negative by the Panbio^TM^ COVID-19 Ag test. The highest viral loads were observed soon after symptom onset, which then gradually decreased but viral loads were very heterogeneous.

## Discussion

This study was designed to evaluate the performance of the Panbio™ COVID-19 Ag test in comparison to RT-PCR, considered the gold-standard diagnostic test for COVID-19. The sensitivity was 84.3% (95% CI 75%‒93.8%) and the specificity was 98.2% (95% CI 96%‒98.8%), when the Ct cutoff used to define positivity was ≤40, similar to results seen in other recent studies.^11^, ^12^, ^13^, ^14^, ^15^

In our study, the agreement beyond chance between the Panbio™ COVID-19 Ag test and RT-PCR decreases as Ct levels increase. In particular, the agreement was 78% for samples with Ct values of ≤ 25 but decreases at higher Cts. Additionally, sensitivity is not significantly different between subjects tested 0 to 11 days after symptom onset due to the high viral load observed in the patients even after 7 days. Following SARS-CoV-2 infection, the virus undergoes a period of incubation during with viral titers usually too low to detect, after which the virus undergoes exponential growth, leading to a peak in viral load and infectivity, followed by declining viral levels and clearance when infectivity is low. For disease control, it is very important to identify both infected individuals who present COVID-19 symptoms and also those who are suspected of infection due to contact with infected individuals.

## Conclusion

The Panbio™ COVID-19 Ag test has sufficient sensitivity and specificity to be used as a tool for the primary screening of such individuals. This is especially true in point-of-care and in patient settings where more elaborate laboratory facilities for RT-PCR are not available.

## Conflicts of interest

The authors declare no conflicts of interest.
